# Characterization and Toxicity Evaluation of Broiler Skin Elastin for Potential Functional Biomaterial in Tissue Engineering

**DOI:** 10.3390/polym14050963

**Published:** 2022-02-28

**Authors:** Nurkhuzaiah Kamaruzaman, Mh Busra Fauzi, Salma Mohamad Yusop

**Affiliations:** 1 Department of Food Sciences, Faculty of Science and Technology, Universiti Kebangsaan Malaysia, Bangi 43600, Malaysia; khuzaiahman@gmail.com; 2 Centre for Tissue Engineering and Regenerative Medicine, Faculty of Medicine, Universiti Kebangsaan Malaysia, Bandar Tun Razak, Kuala Lumpur 56000, Malaysia; fauzibusra@ukm.edu.my

**Keywords:** water-soluble elastin powder, primary skin irritation, toxicity, broiler skin, biomaterial, tissue engineering

## Abstract

Broiler skin, a by-product of poultry processing, has been proven to contain essential elastin, a high-value protein with many applications. The present study reported the extraction of water-soluble elastin from broiler skin by using sodium chloride (NaCl), sodium hydroxide (NaOH), and oxalic acid treatment before freeze-drying. Chemical characterization such as protein and fat content, Fourier-transform infrared (FTIR) spectroscopy, amino acid composition and thermal gravimetric analysis (TGA) were performed and compared with commercial elastin from bovine neck ligament. The resultant elastin’s toxicity was analyzed using an MTT (3-(4,5-dimethylthiazol-2-yl)-2,5-diphenyltetrazolium bromide) tetrazolium assay and primary skin irritation test. Results showed a high quality of the extracted-elastin with the presence of a high amount of proline (6.55 ± 0.40%) and glycine (9.65 ± 0.44%), low amount of hydroxyproline (0.80 ± 0.32%), methionine (2.04 ± 0.05%), and histidine (1.81 ± 0.05%) together with calculated 0.56 isoleucine/leucine ratio. FTIR analysis showed the presence of typical peaks of amide A, B, I, and II for protein with high denaturation temperature around 322.9 °C. The non-toxic effect of the extracted elastin was observed at a concentration lower than 0.5 mg/mL. Therefore, water-soluble elastin powder extracted from broiler skin can be an alternative source of elastin as a biomaterial for tissue engineering applications.

## 1. Introduction

The term “poultry” refers to any of a variety of domesticated birds that humans keep for the purpose of producing eggs, meat, or feathers [1]. The poultry industry is one of the fastest-growing agricultural sectors in the world. With the growth of the poultry industry, it is becoming more challenging to manage the waste it generates [2]. These wastes contribute to one-third of its annual production and should be managed appropriately for environmental reasons and to create high value-added products that can be used in various fields, such as food, pharmaceuticals, and biomedicine [3,4]. Previously, the conversion of animal waste into animal feed and organic fertilizer were standard practices. However, many studies on poultry and other animal by-products have focused on the extraction, isolation, and use of bioactive compounds within those waste materials in downstream processing or other high-end value applications. Proteins hydrolysate, enzymes, and lipids are some biomolecules that can recover from animal by-products [5,6,7]. For instance, it has been shown that poultry viscera are rich in proteolytic enzymes to recover soluble protein that later can be used in the formulation of poultry or fish feed [6]. The processing of the squid industry also generates a lot of by-products such as skin, viscera, head, ink, and beak as potential raw materials to extract elastin [5].

The most common by-products in the carcass of broilers are feathers, skin, bones, and viscera, which account for 26.4% of the live weight of the birds after slaughter [8]. One of the common wastes generated from the broiler chicken industry is the skin. Chicken skin covers almost the entire body of the chicken; thus, millions of tons of chicken skin are generated annually in the worldwide poultry industries as the consumption of poultry meat increases. This chicken skin has been discarded daily due to its high-fat content. Chicken skin is comprised of 22% protein, which can be an excellent source to isolate elastin [9]. Elastin, a protein found in various connective tissues, is essential for blood vessels, lungs, and skin flexibility and support [10,11,12,13]. Ultimately, the body’s elastin production slows down and eventually stops in adulthood and affects the functionality and properties of tissues and organs.

Elastin, in nature, is an insoluble protein due to the high number of covalent crosslinks [14]. As a result of its insolubility in water, the study of elastin’s characteristics and structure has been limited. Therefore, many approaches have been developed to make the insoluble form of elastin become water-soluble by specific treatment using acid, alkaline, or enzymatic hydrolysis [14,15]. Treatment with acids has been frequently employed to hydrolyze elastin as it may retain the majority of the amino acid compositions, particularly acidic amino acids [14]. A soluble form of elastin is highly desirable as analysis and handling of the material can be more practical and convenient. Consequently, access to its bioactivity properties (antioxidative, antihypertensive, etc.) and delivery of its active compound to the body system will be more effective.

Besides the skin, the other elastin sources are from bovine ligamentum nuchae [16], the porcine aorta, rat lung, rabbit lung, and human aorta [17]. The use of elastin from these sources is restricted for a variety of reasons, including practicality, religious prohibitions, and health concerns. In rare cases, bovine elastin has been linked to diseases such as bovine spongiform encephalopathy (BSE), and porcine elastin is severely forbidden in Judaism and Islam. Thus, elastin has also been extracted from marine animals like fish aorta [18] and jumbo squid skin [5]. Properties of elastin could vary depending on the habitat, species, and part of the body it is isolated from, demanding a need for characterization of this protein from different sources. Elastin from broiler chicken skin by-product was first isolated by Nadalian et al. [19]. Several consecutive studies reported the presence of its peptides with antioxidative qualities and as a potential ingredient for cosmetics formulation [9,20]. Understanding elastin’s potential features and applications from various species requires knowledge of its characteristics and bioactive capabilities.

Elastin can be used in various applications, particularly in anti-ageing cosmetics, pharmaceuticals, and tissue regeneration regimens. The use of elastin as biomaterials offers great opportunities for tissue engineering. Researchers have explored using elastin in tissue-engineered skin [21], vascular grafts [22], heart valves [23], cartilage constructs [24], and bone regeneration [25]. For example, biomaterials can incorporate elastin as an insoluble fiber or hydrolyzed elastin; it can also be used in combination with other biopolymers; or it can be used as a block copolymer with other biopolymers. Elastin can also be used in combination with other biopolymers [26]. In vitro and in vivo studies have shown that biomaterials resembling elastin have biological and physical benefits [27,28,29,30]. The capacity to fine-tune elastin compositions has sparked a surge in interest for their use as biomaterials.

Although widely used in tissue engineering, studies on the isolation and characterization of soluble elastin from natural sources (animal) are limited. Besides, these animal-derived materials could cause undesirable host responses upon contact with the skin. As a result, toxicological evaluation and skin reactivity assessment, such as irritation, are required to ensure the safety of customers who may be exposed to the material through intended use, mistreatment, or accidental skin exposure. In addition, the development of this natural biomaterial is crucial and has advantages in clinical applications due to its excellent biocompatibility, biodegradability, functionality, and low immunogenicity [31].

Therefore, the purpose of this study was to determine the chemical properties of the water-soluble elastin powder extracted from broiler skin. Furthermore, evaluation of its biocompatibility to cause skin irritation and toxicity was also carried out for its potential as a biomaterial in tissue engineering applications.

## 2. Materials and Methods

### 2.1. Elastin Extraction and Purification from Broiler Skin

Broiler skin was provided from the Kerabat Processing House Sdn. Bhd, Pedas, Negeri Sembilan, Malaysia. The skin was maintained at –18 °C in the freezer and thawed for about 1 h before usage. To confirm the quality of the extracted elastin, chemical-grade commercial elastin (Sigma-Aldrich, St. Louis, MO, USA), harvested from bovine neck ligament, was used as a benchmark. The extraction of elastin from broiler skin was performed using the method reported by Nadalian et al. [20] and Kamaruzaman et al. [32] with some modification. First, visible fat on broiler skin was removed manually after being thawed. The skin was then suspended in 1 M NaCl for 24 h in a cold room. Next, the homogenate was centrifuged at 13,000× *g* (5804R, Eppendorf, Hamburg, Germany) for 5 min. Subsequently, the pellet was suspended in hot NaOH (0.1 N) for 1 h while being constantly stirred. The residues of NaOH-insoluble material were lyophilized before further analyses. Before freeze-drying, the powder was treated with oxalic acid (0.25 M) for 45 min to facilitate the formation of water-soluble elastin.

### 2.2. Crude Protein Analysis

The crude protein of extracted elastin powder was determined using the Association of Official Analytical Chemists’ standard procedure (AOAC, 2005). The Kjeldahl method determined the nitrogen content using nitrogen to a protein conversion factor of 6.25. The total nitrogen content was calculated using the following equation:

(1) $$\;\mathrm{Nitrogen}\;\left(\%\right)=\frac{\mathrm{mL}\;\mathrm{HCl}\;\left(\mathrm{sample}\right)-\;\mathrm{mL}\;\mathrm{HCl}\;\left(\mathrm{blank}\right)\times N\;\mathrm{HCl}\times 1.4007}{\mathrm{Weight}\;\mathrm{of}\;\mathrm{sample}\;(g)}$$

Crude protein = % N × 6.25(2)

### 2.3. Crude Fat Analysis

According to AOAC Method 960.39 (2005), the Soxhlet method was used to determine the fat content of the extracted elastin powder. The following equation was used to compute the fat content:

(3) $$\%\;\mathrm{fat}\;\mathrm{on}\;\mathrm{dry}\;\mathrm{weight}\;\mathrm{basis}=\frac{g\;\mathrm{of}\;\mathrm{fat}\;\mathrm{in}\;\mathrm{sample}}{g\;\mathrm{of}\;\mathrm{dried}\;\mathrm{sample}}\times 100$$

### 2.4. Functional Group Analysis Using FTIR (Fourier-Transform Infrared Spectroscopy)

The FTIR spectra of extracted elastin powder and commercial elastin were acquired using a universal attenuated total reflectance (ATR) unit on an FTIR spectrophotometer (Perkin Elmer, Waltham, MA, USA). Sample signals were acquired in transmission mode with resolutions of 4 cm^−1^ from 600 to 4000 cm^−1^. The Perkin Elmer Spectrum program was used to graph the FTIR spectra.

### 2.5. Amino Acid Composition

Amino acid analysis was performed according to the method described by Saiga et al. [33] with modification. Elastin powder was hydrolyzed in 6 mol/L HCl for 16 h. The amino acid composition was obtained using high-performance liquid chromatography (HPLC; Waters, Dublin, Ireland), equipped with a Waters 410 Scanning Fluorescence and AccQ Tag column (3.9 × 150 mm). AccQ Tag Eluent A and AccQ Tag Eluent B or 60% acetonitrile acid was used as the mobile phase (flow rate = 1 mL/min). For amino acid quantification, the absorbance measurement was carried out at 248 nm, while the associated fluorescence detector was measured at excitation and emission of 250 nm and 395 nm, respectively.

### 2.6. Thermal Gravimetric Analysis (TGA)

Thermal gravimetric analysis (TGA) was performed using a thermogravimetric analyzer (Shimadzu TGA-50, Shimadzu Corporation, Kyoto, Japan) at the nitrogen flow rate of 40.0 mL/min to determine the thermal stability of the extracted elastin sample. The ramp heat flow was adjusted between 10 and 600 °C/min. The decomposition of sample weight (%) against temperature (°C) was recorded.

### 2.7. MTT (3-(4,5-Dimethylthiazol-2-yl)-2,5-diphenyltetrazolium Bromide Tetrazolium) Assay

This study was performed in compliance with the in vitro safety study following ISO 10993-5:2009 (E). Passage number three (P3) of V79-4 cells (Chinese hamster lung cells; Cricetulus griseus, V79-4, CCL-93™) was grown in tissue culture flasks using DMEM as the growth medium at 37 °C in the CO_2_ incubator (humidified atmosphere of 5% CO_2_ and 95% air). The cells were seeded into a 96-well plate at a seeding density of 10,000 cells/well and incubated at 37 °C for at least 12 h or until 80% confluency was attained. The test substance was tested in triplicate at the concentrations of 0.0625, 0.125, 0.25, 0.5, 1, and 2 mg/mL in a complete growth medium (DMEM). The growth medium was replaced with 200 μL of the test material solution in each well of the 96-well plate containing healthy culture. Following that, the cultures were cultured for 24 h at 37 °C in a CO_2_ incubator. A positive control of hydrogen peroxide at a concentration of 10 mM was used, while a complete growth medium was used as a negative control. A 5 mg/mL MTT solution was added to each well and incubated for 4 h at 37 °C in the CO_2_ incubator. The purple formazan crystals were solubilized in dimethyl sulfoxide (DMSO), and the optical density was determined at 570 nm. The percentage of cell viability was calculated by the following equation:

(4) $$\mathrm{Cell}\;\mathrm{Viability}\;(\%)=\frac{\mathrm{OD}\;\mathrm{of}\;\mathrm{test}\;\mathrm{substance}\;-\;\mathrm{OD}\;\mathrm{blank}\;\mathrm{sample}}{\mathrm{OD}\;\mathrm{negative}\;\mathrm{control}}\times 100$$

### 2.8. Primary Skin Irritation

#### 2.8.1. Preparation of the Application Site

This study was approved by the Universiti Kebangsaan Malaysia Research Ethics Committee (UKMREC) with the approval code UKM PPI/111/8/JEP-2020-453. This study was performed in compliance with the appropriate provision of Consumer Product Safety Commission, Title 16, Chapter II, and Part 1500 and also according to ISO 10993-10:2010(E). Biological evaluation of medical devices—Part 10. The study included only animals without a pre-existing of skin irritation. The dorsal surfaces of six healthy young adult New Zealand albino rabbits were examined (four males and two females). Each side of the rabbit’s dorsal skin was trimmed free of hair to expose a 10 cm by 15 cm surface. Precautions were taken to avoid abrasion and harm to the skin. After skin exposure and before treatment, the animals were re-examined for abnormalities and signs of illness. Six test locations were identified, two for the application of test material and four for controls (for each site, the skin was abraded in one area and left intact in the other).

#### 2.8.2. Preparation of Test Material

A total of 0.5 g of the test material was applied neatly on the test site. Then, absorbent gauze (2.5 cm × 2.5 cm) soaked in normal saline was used as the negative control. Meanwhile, the positive control, i.e., SDS in petroleum jelly, was prepared by spreading 0.5 g on a filter paper at the corresponding control site.

#### 2.8.3. Application of Test Material

The test material and control item were applied to each of the prepared skin’s indicated sites. To limit evaporation and avoid dislocation of test patches, the locations were individually coated with double-layered surgical gauze and wrapped with non-reactive adhesive tape. The rabbits’ entire trunks were then coated in a rubberized material, and they were returned to their cages.

After 24 h of exposure, all patches, including the rubberized cloth, were removed. To remove any residues, the treated areas were gently wiped with a moist clean towel. Individual locations were then scored by four observers using a skin reaction scoring system at 24, and 72 h following patch removal.

#### 2.8.4. Method of Scoring

Skin lesions on each application site were rated and scored according to the Draize scoring system at 24 and 72 h after patch removal as shown in Table 1. The erythema and oedema scores were added together (abraded and unabraded skin included), and the sum of the added scores was divided by four. If the Primary Irritation Index (PII) of the test material is equal to or more than five, it is classified as a primary irritant.

### 2.9. Statistical Analysis

Each analysis was performed in triplicate. A one-way analysis of variance (ANOVA) was used to compare means, and the Duncan range test was used to determine significance at a level of 95% (*p* < 0.05). SPSS was used to conduct the statistical analyses (SPSS 23.0 for windows, SPSS IBM, Chicago, IL, USA).

## 3. Results and Discussion

### 3.1. Yield, Crude Protein, and Fat Composition

**Figure 1:**
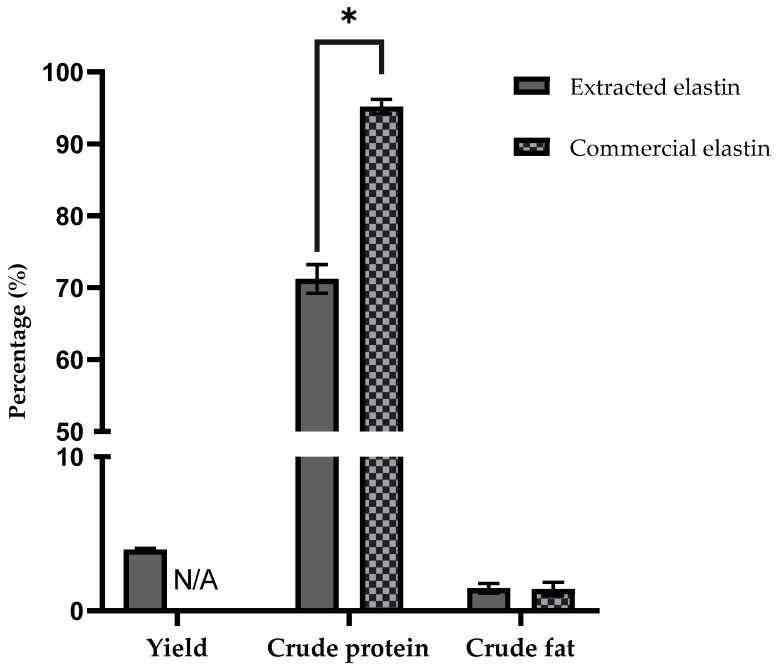
The percentage of yield, crude protein and fat composition of extracted elastin as compared to commercially available elastin from bovine neck ligament. * Represented significant difference (*p* < 0.05).

The results for yield, crude protein and fat composition are shown in Figure 1. This study obtained a 4 ± 0.09% yield of elastin (40 g of elastin/1kg of chicken skin). For comparison, the yield of elastin from jumbo squid’s skin is 1.4% [5], and in the fish bulbus arteriosus, the yield is 34.8% [34]. Extracted elastin consisted of 71.2 ± 1.99% of crude protein and 1.4 ± 0.39% of crude fat. The remaining 27% of the composition is accounted for the moisture and ash content of the extracted elastin. The results showed the effectiveness of the extraction process used in this study as it removed the majority of the fat content and retained more than half of the protein content. Chicken skin is rich in fats [35,36]. Thus, removing this fat content is a challenge as high-fat content can affect the sample’s protein and amino acid compositions. While comparing with the commercial elastin, the crude fat content is not significantly different (1.4 ± 0.48%), however it is significantly higher in terms of its protein content (95.2 ± 1.02%). The same goes while comparing with the protein content of the bulbus arteriosus of yellowtail (*S. quinqueradiata*) fish, which is 93% [34].

High protein contents of species other than the ones used in this study for extracted elastin might be due to the extraction method used. The use of protease in the study by Nakaba et al. [34] improved extractability and high recovery compared to the non-enzymatic procedure used in the present study. The enzyme would improve protein solubility by hydrolyzing it into the solvent; thus, increasing extraction yield [37]. Another difference observed here is that the bulbus arteriosus is the dilated part of the aorta, which are the most elastic arteries in the body and thus rich in elastin fibers. As compared to the skin, elastin accounts for around 2–4% of the dry weight of the skin dermis, whereas collagen accounts for the remainder [38,39]. Therefore, the yield and protein content of extracted elastin is expected to be lower in this present study.

### 3.2. Fourier-Transform Infrared (FTIR) Spectroscopy

**Figure 2:**
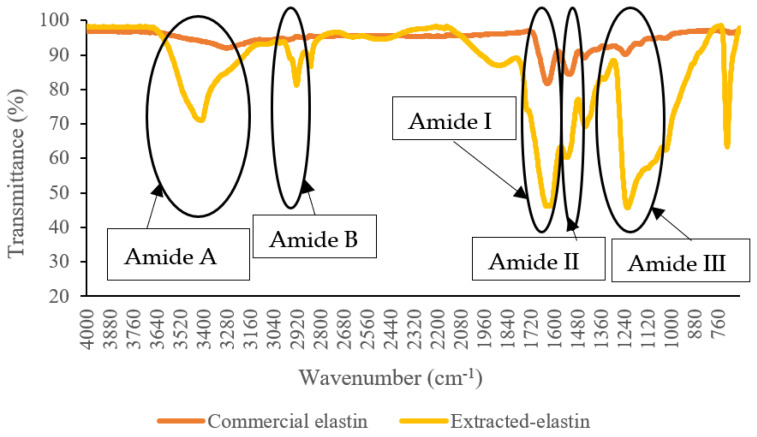
FTIR spectrum analysis of extracted elastin from broiler skin compared with commercially available elastin from bovine neck ligament.

The secondary structure composition of proteins can be determined using FTIR spectroscopy. The FTIR spectra for extracted poultry-based elastin and commercial elastin from bovine neck ligament are shown in Figure 2.

FTIR analysis showed typical peaks of protein, which are amide A, B, I, II and III for both samples. The assignment of wavelength bands in the FTIR spectra of analyzed samples is given in Table 2 and compared with elastin extracted from previously reported studies [5,40]. The 1537 cm^−1^ and 1622 cm^−1^ peaks are directly associated with the α-helix and β-sheet structures, respectively, confirming the secondary structure of the extracted elastin. Furthermore, the absorbance at the higher wavenumbers (1649–1651 cm^−1^, 1540–1550 cm^−1^, and 1290–1320 cm^−1^) predominantly correlates to α-helical structures, whereas the lower wavenumbers (1620–1630 cm^−1^, 1520–1540 cm^−1^, and 1220−1240 cm^−1^) are essentially the characteristic of β-structures [41,42].

The absorbance peak intensities of the extracted elastin were greater than those of the commercial elastin detected in the commercial FTIR spectrum. One could argue that the heterogeneous nature of isolated elastin results in wavelength overlap between identical vibrational functional active groups, hence increasing absorbance intensities. However, as demonstrated in Table 2, elastin from jumbo squid and tannery waste exhibits similar FTIR patterns to extracted and commercial elastin. FTIR technique is a powerful method for a rapid chemical structural characterization of elastin properties from various sources. However, identifying the chemical structural characteristics of elastin from various sources with precision and accuracy is a challenging task. In addition, the wide ranges and slight variations in the IR spectra are highly due to the raw material sources and the pretreatment and extraction techniques utilized.

### 3.3. Amino Acid Composition

The amino acid compositions of the extracted elastin from broiler skin and commercial elastin are summarized in Table 3. Elastin is known to have a very distinctive amino acid composition. The purity of elastin was evaluated by the high glycine levels, alanine, proline, and valine, and low content of polar amino acids (aspartate, glutamate, lysine, histidine, and arginine) [15]. Starcher and Galione [17] reported that elastin in the connective tissue lacks methionine and histidine. Other criteria were also used to discuss elastin purity, such as the low amount of hydroxyproline (due to its relation to collagen) and the estimation of isoleucine/leucine ratio of around 0.35–0.60 [43].

This study reported that glycine and proline were higher for the extracted elastin and lower methionine, histidine, and hydroxyproline. The ratio of isoleucine/leucine was 0.56, which is in range of 0.35–0.60 as mentioned before. These results are in line and have similar patterns with commercial elastin as reported by Yusop et al. [9] and other elastin peptides from previous studies [19,20]. The detection of high levels of methionine and histidine is related to fibrils and microfibril residues of collagen [39]. Therefore, it can be said that the extracted elastin has an elastin property based on the amino acid analysis.

Comparison can also be made with crude elastin extracted from the skin of jumbo squid *Dosidicus gigas* [5]. Elastin from the jumbo squid skin has a slightly higher proline content (8.3%) than the extracted elastin, however, lower glycine content (6.7%). On the other hand, histidine was absent in the jumbo squid’s skin elastin. Nakaba et al. [34] analyzed the amino acid composition in whole and hydrolyzed elastin peptide from the bulbus arteriosus of Pacific yellowtail (*Seriola quiqueradiata*), while Shiratsuchi et al. [18] performed a similar evaluation on bulbus arteriosus of skipjack tuna elastin hydrolysate. In both cases, the glycine and proline levels found in the fish elastin were higher, which was around 40% and 10%, respectively, than those measured in this present study. Variations of amino acid content were observed among different animal species as it is highly related to the protein content of the sample. For example, the protein content of elastin extracted from bulbus arteriosus of fish is 93% compared to only 71% for this study, leading to higher content of each amino acid composition.

Recent data on other poultry or mammalians’ amino acid profiling was scarcely reported. Elastin obtained from pig aorta showed a high percentage of hydrophobic amino acids (Gly, Ala, Val, Pro, leu, and Ile), which accounted for almost 89% of the composition [44]. In addition, in 1976, Starcher and Galione [17] have compared the amino acid analyses of elastins from different tissues of 10 different animal species. For all species, the amino acid distribution was consistent, where a high percentage of Gly, Ala, Val, and Pro were present, which are around 10–30%. Furthermore, elastins from the species studied were known to be deficient in methionine.

### 3.4. Thermal Gravimetric Analysis (TGA)

**Figure 3:**
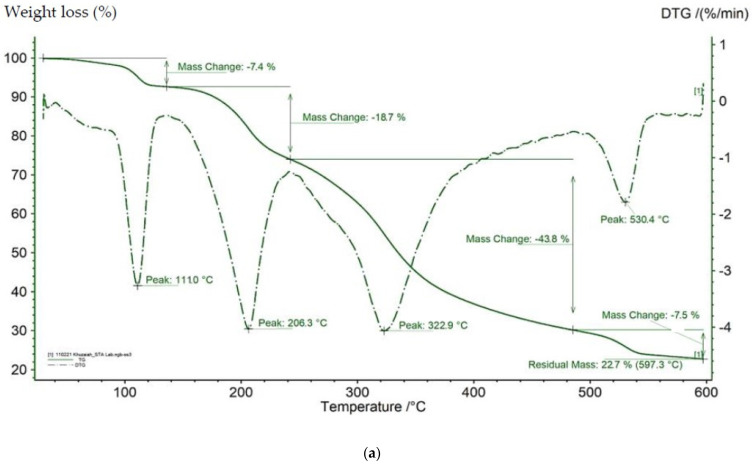

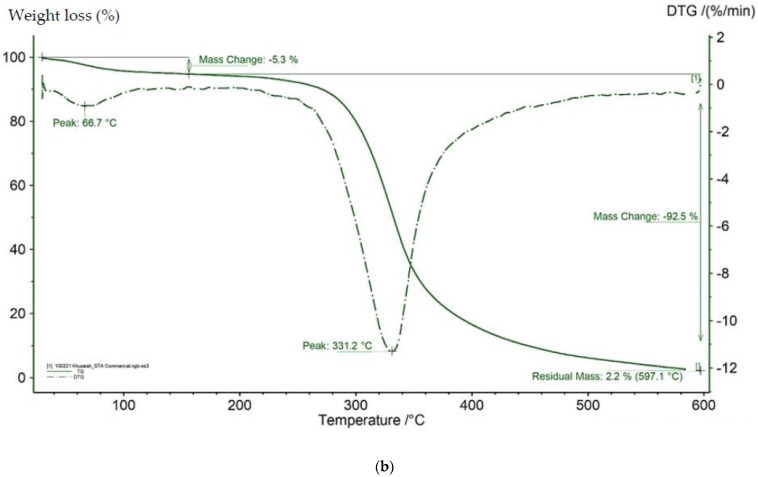
(**a**) Thermal gravimetric analysis (TGA) of extracted elastin from broiler skin (**b**) Thermal gravimetric analysis (TGA) of commercial elastin.

The TGA thermogram of extracted and commercial elastin is shown in Figure 3a,b, with the decomposition peaks in terms of weight loss in percentage as a function of temperature. Based on the TGA thermogram result, extracted elastin shows degradation peaks at four points, whereas commercial elastin has two degradation peaks.

The first decomposition peak of extracted elastin due to removal of moisture and water molecules shows a 7.4% weight loss in the temperature range of about 0–110 °C, while the second degradation peak shows a 18.7% weight loss in the range between 120–240 °C. The maximum elastin decomposition or weight loss is at 43.8%, occurring in the temperature range from 240–340 °C. Finally, the fourth degradation peak shows a 7.5% weight loss in the range of 480–560 °C. On the other hand, commercial elastin from bovine showed only two mass-loss steps, with the maximum percentage of elastin decomposition being 92.5% in the range of 260–360 °C.

The findings show that the elastin that was extracted has good thermal stability, as did the commercial as both samples had maximum weight loss at a high temperature, around 320–330 °C. This property enables elastin to be used as a biomaterial with more resistance to decomposition at high temperatures, providing an advantage for applications where thermal stability is essential [45].

The other proximate components of the extracted elastin, i.e., minerals, might affect the differences in weight loss peaks between the extracted and commercial elastin. Elastin made from tannery waste had six mass-loss steps, where the maximum percentage of elastin decomposition was 32% in the range of 298–367 °C [40].

### 3.5. MTT (3-(4,5-Dimethylthiazol-2-yl)-2,5-diphenyltetrazolium Bromide Tetrazolium) Assay

**Figure 4:**
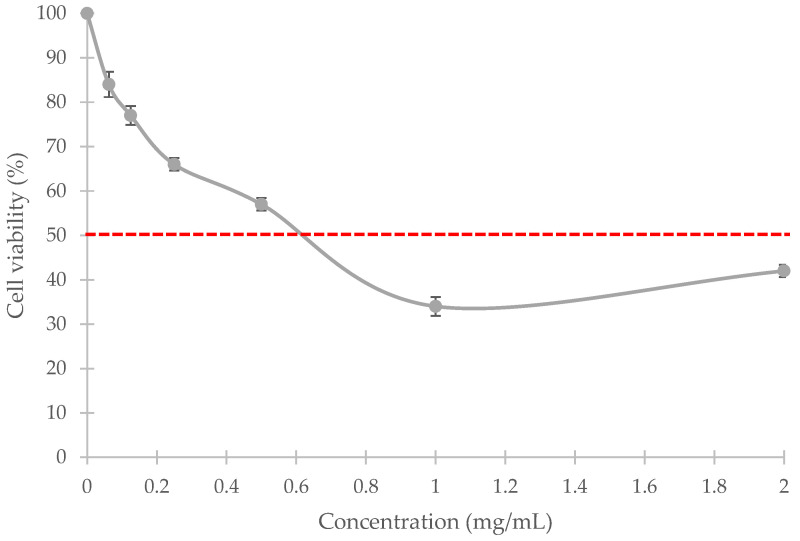
Viability of V79-4 cells at various concentrations of the extracted elastin from broiler skin.

The MTT assay was performed to evaluate the cytotoxic effects of the extracted elastin on V79-4 cells (Chinese hamster lung cells). Figure 4 shows that cell viability decreases with increasing elastin concentrations.

According to the standard procedure, the concentration of a substance that causes more than 50% cell viability is considered non-cytotoxic [46]. The MTT assay indicated that the extracted elastin powder maintained the viability of the cells at ≥50% and is non-toxic at the concentration of ≤0.5 mg/mL in this study.

A biocompatibility study conducted on the extracted elastin from tannery wastes also showed the non-toxic nature of extracted elastin. The selected extraction process did not cause any toxicity development at the concentration up to 0.2 mg/mL [40], which is similar to the non-toxic concentration limit in the present study. Elastin hydrolysate from the bulbus arteriosus of skipjack tuna significantly enhanced fibroblast proliferation from 1.10- to 1.19-fold after 96 h at 5 × 10^−6^–0.005 mg/mL [18]. Both studies showed that an adequate amount of elastin concentration could enhance cell proliferation and be non-toxic to the cells. Information from MTT assay is essential, especially for biomaterial development for tissue engineering, as the maximum elastin concentration that causes toxicity to the cells could be determined.

### 3.6. Primary Skin Irritation Test

Primary skin irritation was assessed using the Draize score to determine the extracted elastin’s potential to irritate following a single topical application on rabbit skin [47]. Prior to patch application, all rabbits appeared to be healthy and active, with no evidence of gross toxicity, adverse pharmacological effects, or abnormal behavior. However, throughout the 72-h observation period, erythema and edoema were observed on the majority of the abraded and intact skin treated with the extracted elastin. According to Table 4, the Primary Irritation Index (PII) of the extracted elastin powder is 5.54. Erythema and edema are also present on some test sites treated with negative control, with a PII of 0.54. Meanwhile, cutaneous reactions at the contact sites with the positive control (SDS in petroleum jelly) produced erythema and edema at 24, and 72 h post-treatment. The PII of positive control is 7.29.

After subtracting the negative control index, the extracted elastin’s PII is 5.00. As a result, the extracted elastin is classified as a primary irritant. However, as the intended use of the extracted elastin is as a biomaterial for tissue engineering, the materials should be highly safe. They should not induce any inflammatory or allergic reactions in humans. Irritation of the skin happened as 100% concentration (0.5 g) of the test material was applied on the test site for this test, which was relatively high and thus irritated the skin.

## 4. Conclusions

The purification process of elastin can be complex due to its insolubility. This study highlighted the possibility of isolating elastin from poultry by-products from broiler chicken skin. We also find it a necessary step to convert it into a water-soluble form to facilitate its use and associate it with other materials.

Characterization works showed that water-soluble elastin was successfully extracted from broiler chicken skin with a yield of 4%. The thermal stability of extracted elastin was relatively higher (322.9 °C), indicating its potential as a thermal stable biomaterial at high temperatures. The FTIR analysis indicated that the extracted elastin possessed the secondary structure for proteins’ α-helix and β-strand. As elastin powder was reported as a potential antioxidative peptide in previous studies, a toxicological evaluation of the extracted elastin has been conducted to ensure its safety in various industries, especially in tissue engineering. The non-toxic effect was observed at a concentration lower than 0.5 mg/mL, which indicates a safe level to be used. Future studies should shed more light on the construct and characterize this extracted elastin as a functional biomaterial both in vitro and in vivo to serve its purposes as a replacement for the damaged elastin fibers or promote the synthesis of elastin.

## Figures and Tables

**Table 1:** Scoring system for skin reaction.

Reactions	Description	Score
Erythema (E)	Erythema and eschar formation	
	No erythema	0
	Very slight erythema (barely perceptible)	1
	Well-defined erythema	2
	Moderate to severe erythema	3
	Severe erythema (beet redness) to slight eschar formations (injuries in depth)	4
Oedema (O)	Oedema formation	
	No oedema	0
	Very slight oedema (barely perceptible)	1
	Slight oedema (edges of the area well defined by definite raising)	2
	Moderate oedema (raised approximately 1 mm)	3
	Severe oedema (raised more than 1 mm and extending beyond the area of exposure)	4

**Table 2:** Comparison of band assignment wavelength of FTIR spectrum of elastin extracted from different sources.

Band Assignment	Band Position (cm^−1^)			
	Extracted elastin from broiler skin	Commercial elastin from bovine neck ligament	Elastin from tannery wastes (Data from Yoseph et al. 2020 [40])	Elastin from Jumbo squid (Data from Ramírez-Guerra et al. 2019 [5])
Amide A (N-H stretching)	3411	3283	3300	3270
Amide B (CH2 asymmetrical stretching)	2923	2969	2925	2950
Amide I (C=O stretching)	1622	1634	1694	1633
Amide II (N-H deformation)	1537	1520	1300	1533

**Table 3:** Amino acid composition (%) of the extracted elastin from broiler skin and commercial elastin from bovine neck ligament analyzed by High-Performance Liquid Chromatography (HPLC).

Amino Acid	Amino Acid Composition (%)	
	Extracted Elastin from Broiler Skin	Commercial Elastin
Aspartic acid	8.82 ± 0.14 ^c^	7.13 ± 0.05 ^d^
Serine	2.04 ± 0.01 ^kl^	2.31 ± 0.01 ^h^
Glycine	9.65 ± 0.44 ^b^	20.40 ± 0.38 ^a^
Glutamate	13.27 ± 0.20 ^a^	2.49 ± 0.13 ^h^
Histidine	1.81 ± 0.05 ^mn^	0.94 ± 0.03 ^ij^
Arginine	4.77 ± 0.19 ^f^	4.65 ± 0.16 ^f^
Threonine	1.14 ± 0.05 ^n^	2.38 ± 0.14 ^h^
Alanine	4.11 ± 0.09 ^fg^	8.51 ± 0.22 ^c^
Proline	6.55 ± 0.40 ^d^	9.04 ± 0.34 ^b^
Tyrosine	2.25 ± 0.12 ^jk^	2.13 ± 0.03 ^h^
Valine	3.83 ± 0.10 ^h^	9.03 ± 0.18 ^b^
Methionine	2.04 ± 0.05 ^kl^	1.15 ± 0.03 ^i^
Lysine	5.15 ± 0.09 ^e^	4.49 ± 0.03 ^f^
Isoleucine	1.92 ± 0.10 ^m^	3.90 ± 0.09 ^g^
Leucine	2.90 ± 0.13 ^j^	6.69 ± 0.11 ^e^
Phenylalanine	3.28 ± 0.08 ^hi^	7.49 ± 0.17 ^d^
Hydroxyproline	0.80 ± 0.32 ^o^	0.74 ± 0.35 ^j^

^a–o^ Mean in the same column without a common superscript letter differ significantly (*p* < 0.05).

**Table 4:** Primary Irritation Indices of extracted elastin powder, negative control, and positive control.

Skin Reactions	Exposure Time (Hours)	Evaluation Values (Average for 6 Rabbits)		
		Extracted Elastin Site	Negative Control Site	Positive Control Site
Erythema and Eschar Formation				
Non abraded skin	24	3.67	0.00	3.83
Non abraded skin	72	1.67	0.00	3.83
Abraded skin	24	4.00	0.33	3.83
Abraded skin	72	3.33	1.17	3.50
Subtotal		12.67	1.50	14.99
Oedema Formation				
Non abraded skin	24	2.83	0.00	3.83
Non abraded skin	72	0.50	0.00	3.83
Abraded skin	24	3.33	0.33	3.17
Abraded skin	72	2.83	0.33	3.33
Subtotal		9.49	0.66	14.16
Total *		22.16	2.16	29.15
Primary Skin Irritation Index		5.54	0.54	7.29

* The evaluation value for each site was divided by 4 (2 scoring intervals × 2 sites).

## Data Availability

Not applicable.
